# Case report: A novel high‐dose intravenous immunoglobulin preparation for the treatment of severe pemphigus vulgaris failing standard therapy

**DOI:** 10.1111/1346-8138.17475

**Published:** 2024-09-30

**Authors:** Nadine Wiedenmayer, Katharina Hogrefe, Silvia Mihalceanu, Julia K. Winkler, Alexander H. Enk

**Affiliations:** ^1^ Department of Dermatology University Hospital Heidelberg Heidelberg Germany

**Keywords:** adverse effects, autoimmune bullous dermatoses, intravenous immunoglobulins, IVIg, pemphigus vulgaris

## Abstract

Pemphigus vulgaris (PV) is a severe autoimmune bullous dermatosis that is characterized by autoantibodies against epidermal adhesion proteins causing painful mucosal and skin blistering. Standard treatments for PV include corticosteroids, steroid‐sparing immunosuppressants, or intravenous monoclonal anti–CD20‐antibody therapy. The European guidelines suggest high‐dose intravenous immunoglobulin (IVIg) therapy as a promising approach for severe or treatment‐resistant cases. We report on a 65‐year‐old woman with severe and recurrent disease who achieved long‐term disease stabilization with IVIg treatment. Because of recurrent fatigue and headache, the patient was switched to an alternative IVIg preparation with a novel manufacturing process, thus ensuring high purity and better tolerability. We observed excellent efficacy, yet side effects remained largely unchanged. Further studies are necessary to evaluate the long‐term efficacy and tolerability of this new IVIg preparation.

## 1 INTRODUCTION

Pemphigus vulgaris (PV) is one of the most severe conditions within the group of autoimmune bullous dermatoses (AIBDs). PV is caused by antibodies directed against desmogleins (Dsg)—desmosomal adhesion proteins that ensure epidermal integrity.^1^, ^2^ Histopathology reveals intraepidermal cleft formation, direct immunofluorescence reticular deposition of IgG, and C3c in the epidermis. Destruction of intracellular connections leads to acantholysis, resulting in blistering formation. These blisters rupture easily, leading to erosions that can result in severe infections. When the oral mucosa is involved, severe malnutrition may occur.^3^ Standard treatment involves systemic steroids administered alone or in combination with other immunosuppressants or rituximab.^4^

For severe cases of PV or in cases of inadequate response to standard treatment, intravenous immunoglobulin (IVIg) therapy has become a promising treatment approach.^5^ IVIgs are antibodies (IgG) pooled from the serum of healthy blood and plasma donors that affect the innate and adaptive immune system leading to effective suppression of the dysregulated immune response. Mechanisms of action comprise reduction of tissue degradation through inhibition of the complement cascade and modulation of the cytokine, phagocyte, and dendritic cell activity. Moreover, IVIg therapy targets circulating autoantibodies through mechanisms that reduce their half‐life, neutralize them, and inhibit their production. Clinical, serological, and immunopathological remission is achieved through distinct modulation of T‐ and B‐cell populations, leading to a tolerance‐associated polarization of the immune system.^6^, ^7^ Concerning PV, there is growing evidence of IVIg effectivity in restoring dysfunctional immune regulation.^8^ The recommended dosage of IVIg is 2 g/kg of body weight distributed over 2 to 5 days every 4 weeks.^9^ A wide range of IVIg preparations is available, all demonstrating nearly identical efficacy. The manufacturing process may differ, leading to slightly distinct safety and tolerability profiles.^10^

There is still a paucity of knowledge regarding the optimal IVIg preparation for the individual patient. Existing recommendations suggest a concise evaluation of preexisting conditions, such as chronic organ disorders or risk of thromboembolic events (e.g. sugar‐free preparations for renal insufficiency).^10^ Relevant criteria for selecting a particular IVIg need to be defined.

Here, we report on a patient with ongoing remission of PV upon receiving long‐term IVIg therapy who underwent a switch of IVIg preparation because of recurrent fatigue and headache.

## 2 CASE REPORT

**FIGURE 1:**
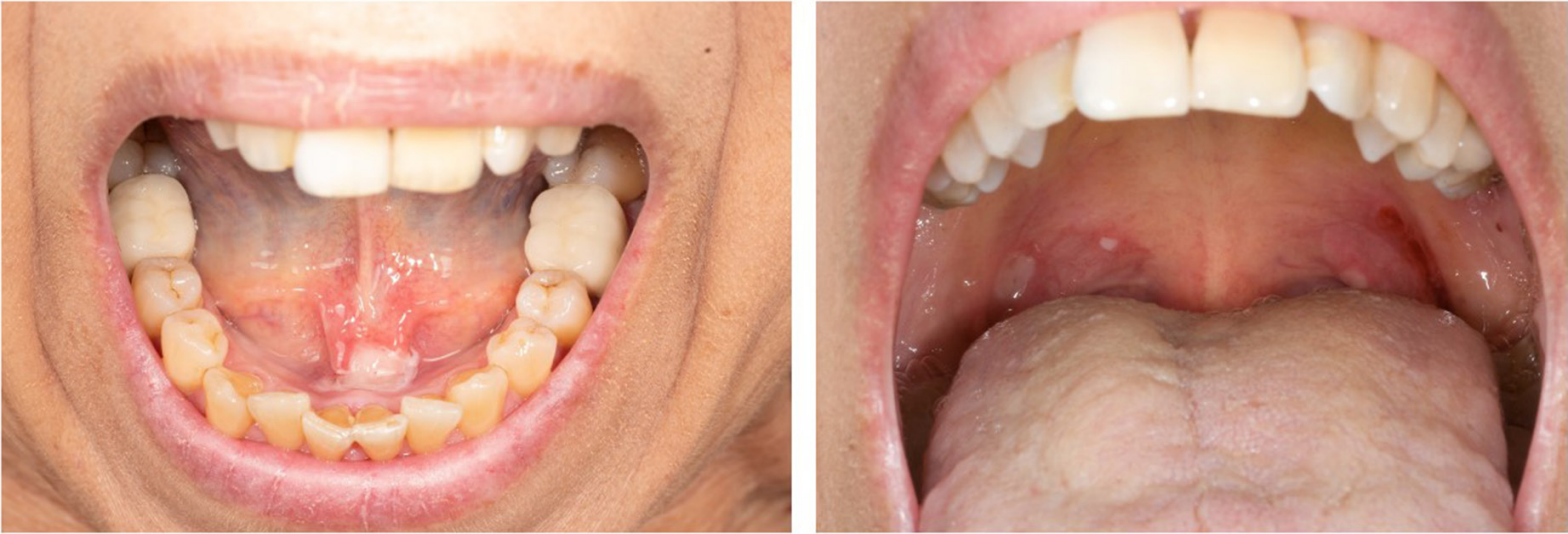
Extensive oral lesions in patient with PV prior to treatment start.

We present the case of a 65‐year‐old woman who first presented to our dermatology department in 2007 with recurrent painful erosions of the oral mucosa that caused difficulty in swallowing and a significant reduction in general condition. Histology and serology led to the diagnosis of PV. Initially, systemic therapy with mycophenolate mofetil 3 g/day and prednisolone 150 mg/day (2 mg/kg of body weight per day) was initiated, which resulted in a healing of lesions of the oral mucosa (Figure 1). Although immunosuppressive therapy was gradually reduced, the patient reported hair loss, depressive mood, and palpitations. Because of multiple recurrences requiring further high‐dose steroid therapy, IVIg treatment (Intratect 100 g/L Biotest AG, 2 g/kg of body weight distributed over 2 days every 4 weeks) was initiated in 2010, leading to improvement and stabilization of the skin lesions for several years. Despite ongoing IVIg treatment and concomitant immunosuppressive therapy with prednisolone 5 mg/day, recurrence in 2015 necessitated further escalation of treatment. A reinitiation of therapy with mycophenolate mofetil 3 g/day and prednisolone 150 mg/day led to significant improvement of the skin condition. To achieve long‐term disease control, rituximab was administered additionally to ongoing IVIg, mycophenolate mofetil 3 g/day, and prednisolone 80 mg/day starting in May 2015. The patient received a total of 10 cycles of rituximab at a dosage of 375 mg/m^2^ of body surface area per cycle over a total period of 21 weeks. Stabilization was reached and immunosuppression was gradually reduced; however, some erosions occurred recurrently, and elevated antibody titers were detected serologically. Therefore, two further cycles of rituximab at a fixed dose of 1000 mg per cycle were administered at an interval of 4 weeks, in addition to the current therapy with IVIg, mycophenolate mofetil 2 g/day, and prednisolone 7.5 mg/day in January and February 2021. Long‐lasting stabilization of the skin lesions was finally achieved with continued IVIg therapy (Intratect 100 g/L Biotest AG, 2 g/kg of body weight distributed over 2 days every 4 weeks). The patient, however, complained about headaches and fatigue occurring after IVIg administration, persisting for up to a week. These are common side effects of IVIg therapy. Hence, in September 2023, therapy was switched to a new immunoglobulin preparation (Yimmugo 100 g/L Biotest AG, 2 g/kg of body weight distributed over 2 days every 4 weeks). We were able to observe good efficacy of therapy with the new IVIg. Concomitant immunosuppression with mycophenolate mofetil and prednisolone could be gradually reduced over the last years and finally discontinued in April 2024. However, the patient continues to report headaches and fatigue after the infusion, despite premedication with oral paracetamol 1 g. The symptoms are roughly comparable to those after administration of the previous IVIg.

## 3 DISCUSSION

IVIgs are therapeutic preparations containing pooled human IgG from thousands of donors. To meet the high quality and safety standards required by the regulatory authorities, these formulations undergo a sophisticated manufacturing process that achieves a highly purified product while preserving structure and function of the IgG antibodies. This complex multistep workflow starts with epidemiological donor screening and plasma collection, followed by product quarantine and validated virus elimination techniques, as well as minimization of IgA concentration.^11^ Commercially available IVIg formulations achieve a purity of at least 95% but vary in terms of the excipient composition comprising IgA, IgM, stabilizing agents, salts, as well as traces of buffers, solvents, and detergents. The most common stabilizing agents include sugars such as sucrose, glucose, maltose, D‐sorbitol, and proteins such as L‐proline or glycine.^12^

IVIgs are used to treat a wide range of autoimmune and inflammatory conditions such as primary and secondary immunodeficiencies, primary immune thrombocytopenia, Kawasaki disease, and neuromuscular conditions.^13^ IVIgs have proven to be particularly effective for dermatological diseases such as dermatomyositis, lupus erythematosus, systemic vasculitis, scleromyxedema, and autoimmune blistering diseases.^5^ IVIgs have proven effective in severe cases of PV that have failed standard therapy and can be regarded as a secondary or tertiary treatment option with a high efficacy, high level of evidence, and overall good tolerability.^14^ Rare side effects include thromboembolic events, hemolytic anemia, renal insufficiency, and aseptic meningitis. In addition, anaphylactic reactions may occur but can be controlled with appropriate medication. The most common side effects are headache, fatigue, nausea, flushing, and fever, which are described in about 10% of patients.^10^ These may be controlled by adjusting the infusion rate, increasing fluid intake, or a premedication with painkillers, antipyretics, antihistamines, or corticosteroids.^10^ Nevertheless, side effects reduce quality of life, differ between patients, and may be associated with different IVIg preparations.

As the landscape of commercially available IVIg formulations expands, efforts are being made towards improving safety and tolerability profiles, starting with innovations of the manufacturing process. Yimmugo, a highly purified, sugar‐free 10% IVIg preparation, underwent optimized manufacturing steps to reduce the risk of adverse reactions. Key improvements have been made to improve tolerability. Notably, the use of efficient but gentle vibromixing has minimized shear stress on the proteins, thereby reducing the potential for protein damage. Moreover, the removal of the complement system activator properdin by an additional chromatography step resulted in reduced levels of anticomplementary, thrombogenic activity.^15^ Compared with other immunoglobulin preparations including Intratect, which was initially used in our patient, Yimmugo stands out because of its low level of protein impurities such as the content of IgA and IgM, complement system activators, as well as coagulation factors that are related to thromboembolic events and antibodies related to hemolytic events.^15^

In conclusion IVIg therapy resulted in significant improvement in our patient's disease activity. However, she reported headaches and fatigue immediately following the infusion of IVIg. Hence, we changed the IVIg preparation to a new IVIg that employs a different manufacturing process. At the present time, we report excellent effectiveness. Comparing the severity of subjective symptoms such as headaches and fatigue remains challenging. Further studies and real‐life experience are important to assess side effects of different IVIg preparations.

## FUNDING INFORMATION

This study was not supported by any sponsor or funder.

## CONFLICT OF INTEREST STATEMENT

A.H.E. received advisory board honoraria, consultancy fees, and support for this publication from Biotest AG. J.K.W. received honoraria und travel support from Biotest AG. N.W., K.H., and S.M. declare no competing interests.

## ETHICAL APPROVAL

Ethical approval was not required for this case report, in accordance with local and national guidelines.

## Data Availability

All data generated or analyzed during this study are included in this article. Further inquiries can be directed to the corresponding author.

## References

[1] Egami S , Yamagami J , Amagai M . Autoimmune bullous skin diseases, pemphigus and pemphigoid. J Allergy Clin Immunol. 2020;145:1031–1047. 32272980 10.1016/j.jaci.2020.02.013

[2] Saschenbrecker S , Karl I , Komorowski L , Probst C , Dähnrich C , Fechner K , et al. Serological diagnosis of autoimmune bullous skin diseases. Front Immunol. 2019;10:1974. 31552014 10.3389/fimmu.2019.01974 PMC6736620

[3] Hsu DY , Brieva J , Sinha AA , Langan SM , Silverberg JI . Comorbidities and inpatient mortality for pemphigus in the U.S.A. Br J Dermatol. 2016;174:1290–1298. 26864457 10.1111/bjd.14463

[4] Joly P , Horvath B , Patsatsi Α , Uzun S , Bech R , Beissert S , et al. Updated S2K guidelines on the management of pemphigus vulgaris and foliaceus initiated by the european academy of dermatology and venereology (EADV). J Eur Acad Dermatol Venereol. 2020;34:1900–1913. 32830877 10.1111/jdv.16752

[5] Hoffmann JH , Enk AH . High‐dose intravenous immunoglobulin in skin autoimmune disease. Front Immunol. 2019;10:1090. 31244821 10.3389/fimmu.2019.01090 PMC6579842

[6] Durandy A , Kaveri SV , Kuijpers TW , Basta M , Miescher S , Ravetch JV , et al. Intravenous immunoglobulins—understanding properties and mechanisms. Clin Exp Immunol. 2009;158:2–13. 19883419 10.1111/j.1365-2249.2009.04022.x PMC2801035

[7] Hudemann C , Hoffmann J , Schmidt E , Hertl M , Eming R . T regulatory cell‐associated tolerance induction by high‐dose immunoglobulins in an HLA‐transgenic mouse model of pemphigus. Cells. 2023;12:1340. 37174740 10.3390/cells12091340 PMC10177252

[8] Hamadah I , Chisti MA , Haider M , Binamer Y , Alajlan S , Aleyouni Y , et al. Rituximab/IVIG in pemphigus – a 10‐year study with a long follow‐up. J Dermatolog Treat. 2019;30:170–175. 29889591 10.1080/09546634.2018.1484873

[9] Svecova D . IVIG therapy in pemphigus vulgaris has corticosteroid‐sparing and immunomodulatory effects. Australas J Dermatol. 2016;57:141–144. 26581165 10.1111/ajd.12422

[10] Cherin P , Marie I , Michallet M , Pelus E , Dantal J , Crave JC , et al. Management of adverse events in the treatment of patients with immunoglobulin therapy: a review of evidence. Autoimmun Rev. 2016;15:71–81. 26384525 10.1016/j.autrev.2015.09.002

[11] Barahona Afonso AF , João CM . The production processes and biological effects of intravenous immunoglobulin. Biomol Ther. 2016;6:15. 10.3390/biom6010015 PMC4808809 27005671

[12] Prins C , Gelfand EW , French LE . Intravenous immunoglobulin: properties, mode of action and practical use in dermatology. Acta Derm Venereol. 2007;87:206–218. 17533485 10.2340/00015555-0249

[13] European Medicines Agency . Guideline on core SmPC for human normal immunoglobulin for intravenous administration (IVIg) – Rev 6. 2021 Reference Number: EMA/CHMP/BPWP/94038/2007 Rev. 6 Corr. Available at: https://www.ema.europa.eu/en/documents/scientific‐guideline/guideline‐core‐smpc‐human‐normal‐immunoglobulin‐intravenous‐administration‐ivig‐rev‐6_en.pdf

[14] Amagai M , Ikeda S , Shimizu H , Iizuka H , Hanada K , Aiba S , et al. A randomized double‐blind trial of intravenous immunoglobulin for pemphigus. J Am Acad Dermatol. 2009;60:595–603. 19293008 10.1016/j.jaad.2008.09.052

[15] Duellberg C , Hannappel A , Kistner S , Maneg O . Biochemical characterization of a new 10% IVIG preparation [IgG next generation (BT595)/Yimmugo(®)] obtained from a manufacturing process preserving IgA/IgM potential of human plasma. Drugs R D. 2023;23:245–255. 37466834 10.1007/s40268-023-00430-w PMC10439088

